# Iranian high risk regions due to esophageal cancer: spatial analysis of cancer registry data 

**Published:** 2018

**Authors:** Hadis Najafimehr, Sara Ashtari, Mohamad Amin Pourhoseingholi, Luca Busani

**Affiliations:** 1 *Gastroenterology and Liver Diseases Research Center, Research Institute for Gastroenterology and Liver Diseases, Shahid Beheshti University of Medical Sciences, Tehran, Iran*; 2 *Basic and Molecular Epidemiology of Gastrointestinal Disorders Research Center, Research Institute for Gastroenterology and Liver Diseases, Shahid Beheshti University of Medical Sciences, Tehran, Iran*; 3 *Department of Infectious Diseases, Instituto Superiore di Sanità, Roma, Italy*

**Keywords:** Esophageal cancer, Relative risks, Bayesian model, Iran

## Abstract

**Aim:**

The aim of this study was to identify the esophageal cancer (EC) high risk regions to evaluate changes of relative risks (RRs) for both genders by time in Iranian provinces.

**Background:**

EC is one of the public health problems in Iran. In spite of this fact, there is not comprehensive study estimating RRs across the Iranian provinces.

**Methods:**

In this cross-sectional study the data for EC cases were extracted from Ministry of Health and Medical Education (MOHME) including 30 provinces from 2004 to 2010. For estimating the model parameters, we used Bayesian approach by regarding spatial correlations of adjacent provinces.

**Results:**

The Northern half of Iran has high risk and other half has low risk. During the time, the range of RRs has decreased for both gender and also the dispersion of EC is decreasing for women but nearly is fixed for men.

**Conclusion:**

While RR has declined during the study, focusing on the Northern half of Iran as high risk regions is a considerable fact for policymakers.

## Introduction

 Esophageal Cancer (EC) is the eighth common cancer and also is the sixth cause of death in Iran (1). Previous studies on common cancers showed that EC has covered one third of all digestive cancers in Iran and its prevalence in men is more than the women (2). In recent years the mortality rate of this cancer has dramatically increased (3, 4). 

It is clear that the cancer incidence due to effects of the environmental factors is not equal in the different geographical regions (5). Previous researches revealed that the distribution of EC in Iran is not homogeneous and differs for different regions and cultures. In Northeast, the Age standardized rate (ASR) of this cancer is about 100 ( per 100000 populations ) and in Northwest is about 30 ( per 100000 populations) while, at the same time there are regions by ASR less than 3 (per 100000 populations) (6).

**Figure 1:**
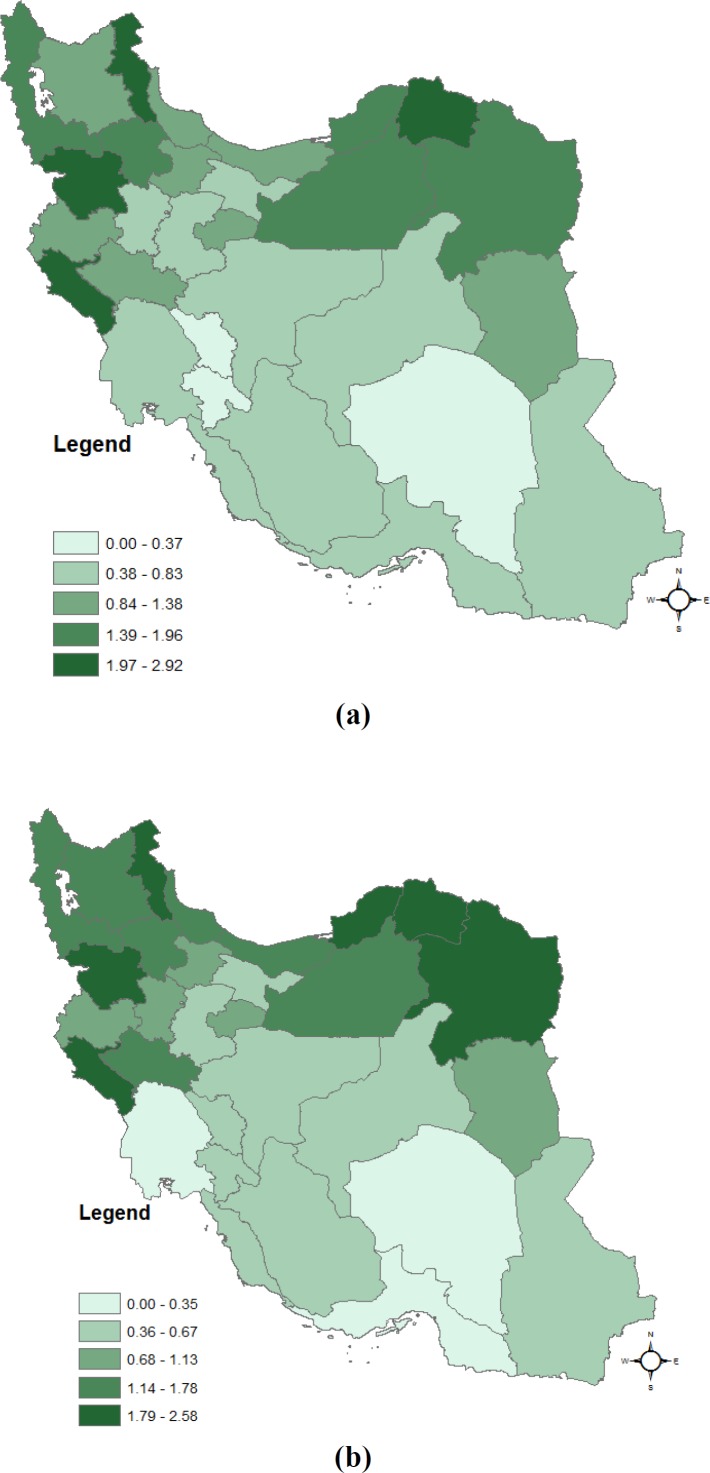
Spatial pattern of Esophageal Cancer Relative Risk in women (a) and men (b) in Iran, 2004-2010

**Figure 2:**
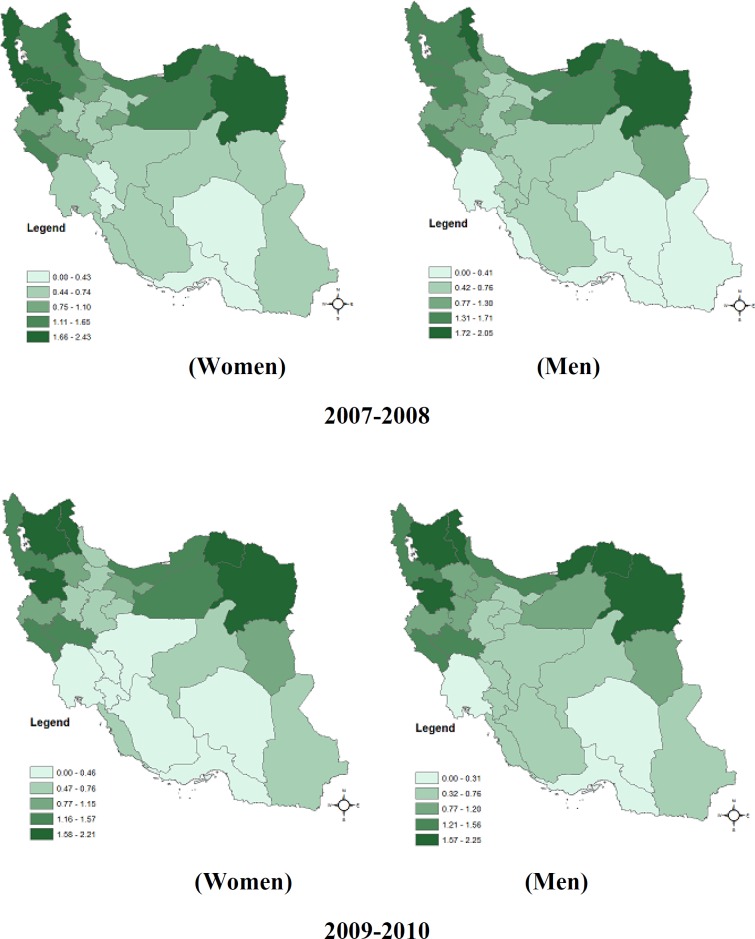
Spatial pattern of esophageal cancer relative risk in women and men in Iran; 2004-2010

As it is clear, mapping of the disease is a useful method that helps to determine the distribution of the disease or its geographical situation. By mapping, it may measure an exact aspect of the disease, which leads to the identification of high risk or at risk places. In addition, it is a useful tool for prediction of disease trend and synergetic factors, as well as investigation of disease etiology (7). In recent years, in order to surveillance of EC spread, several studies have been conducted to map EC distribution in some countries, such as China and Africa (8, 9). In Iran, Chamanpara* et al.* performed the model for geographical variation for EC in Golestan province (in the North part) (10), also in Marzoni *et al.* research reported the EC pattern with the aid of adjusted model (11). Ahari *et al*., study through the geographical information system showed the relationship between EC and climatic factors (12). These studies were conducted only in a limited sections of the country or had used the methods with low level of precision. In current study we performed a nationwide research by using registry data to identify the EC high risk regions in Iranian provinces. For the mapping, we are going to employ the spatial correlation due to adjacent regions. 

## Methods

***Data collection***

In this cross-sectional study, the data for EC cases registered in National Cancer Registry System were extracted from Iranian Ministry of Health and Medical Education (MOHME). This data is collected according to ICD10: C15 cods and are including 30 Iranian provinces from 2004-2010. Hospital records and records from diagnostic departments, in particular histopathology, are the principal sources of cancer registries (13). The world health organization (WHO) has recommended all government to create and manage the cancer data for control programs through the registry system. The history of cancer registry in Iran was initiated from 1995 by establishing the Cancer Institute at University of Tehran (14). The quality of data depend on the complete coverage of all cases with the exact information (15). 

Information of at risk population was obtained from the Population and Housing Census data by the statistical center of Iran (SCI) (16). The ASR for EC was calculated according to direct standardization method and the standard population in WHO 2000 for both genders and 10 years old grouping (17). 

***Statistical analysis***

Since the outcome was a countable variable we assumed that it has a Poisson distribution. For spatial analysis of outcome, it is supposed that the number of observed new cases has a Poisson ($\gamma_{i}E_{i}$) distribution (i=1,…,n). Where $\gamma_{i}$ is RR, $E_{i}$ is the expected number of cases which was calculated with the aid of internal standardized method. The random effect model was used for RRs estimating and its formula is as follow:

$\mathrm{Log}\left(\gamma_{i}\right)=\alpha +u_{i}+\delta_{i};i=1,\ldots ,n$

Where α is the overall effect; $u_{i}$ is the non-spatial (uncorrelated heterogenuse) term; and $\delta_{i}$ is the spatial (correlated heterogeneous) term (18). For$\delta_{i}$, the Conditional Auto Regressive (CAR) model was used for prior distribution in scope of Bayesian method. The spatial component in the present model, by using CAR model, will regard the effect of adjacent points at every locality. The prior distribution for parameters is as follow: αα ~Normal(0,0.001), $u_{i}$ ~Normal (0, $\sigma^{2}$) where $\sigma^{2}\mathrm{\sim gamma}(0.01,0.01)$ (19). We utilized the Marcov Chain Monte Carlo (MCMC) technics by 100000 iterative samples. For analyses of data the WINBUGS program was used and all maps were produced with ArcGIS10. 

## Results

A sum of 21896 EC cases has been found through 2004-2010 in Iran. The numbers of incidence cases and the ASR for EC in each year are presented in table 1. 

In our study, the number of expected cases in each sex was calculated with the aid of internal standardized method. The range of RRs for women were between 0.3, 2.92. In women population, the maximum RR of EC is 2.92 and belongs to Ilam (%95 CI: (2.66, 3.41)). The pattern of the RR estimation is shown as Figure 1(a). According to the map, Ardabil, North Khorasan and West Azerbayejan (Northern part of Iran) provinces, were as high risk regions. 

 In the men population, the maximum RR is 2.60 and belongs to North Khorasan (%95 CI: (2.29, 2.94)). Ardabil, Khorasan Razavi and Golestan provinces (Northern part of Iran) had high RRs, respectively. RR range was between 0.24, 2.58 for women. The pattern of RRs for men is shown as figure 1(b).

The expansion of EC distribution for each sex was evaluated by time (at the three times duration). During 2004-2006, in women population, RRs were varied from 0.06 to 4.79. In men population were 0.06 to 4.70. During 2007-2008, RRs were 0.35 to 2.43 for women and for men were 0.27 to 2.05. Since 2009-2010, RRs were 0.3 to 2.21 and 0.22 to 2.25 for women and men, respectively. Figure 2 is indicating the pattern of RRs for comparison. It seems the EC spread has been decreased by time specially, for women. The North part provinces remained as high risk areas.

## Discussion

One of the most common cancer in the Asia including Iran is EC (20). Table 2 is showing ranking of EC among 10 Iranian common cancers based on reports extracted from Iranian MOHME (21). This comparison indicates the EC rank for women is maintained partly fixed by time and unfortunately, EC is the fifth cancer. For men, the trend is almost improving and the rank is increasing.

**Table 1:** The number of cases and ASR (per 100000) for EC in Iran

	women		men	
year	Number (percent)	ASR	Number (percent)	ASR
2004	1405 (49%)	4.9	1448 (51%)	4.6
2005	1417 (48%)	5.4	1533 (52%)	5
2006	1390 (49%)	6.2	1447 (51%)	5.8
2007	1448 (47%)	6.07	1611 (53%)	6.25
2008	1380 (45%)	6.14	1688 (55 %)	6.16
2009	1750 (47%)	7.77	1964 (53%)	7.66
2010	1533 (46%)	5.88	1823 (54%)	6.15

Several studies were carried out on ASR of EC in Iran. Almasi *et al.* in a study on checking mortality and morbidity due to cancers pointed that at 2012, the ASR of all cancers was equal to 127.7 per 100000 and for EC was 8.6. Also it has been indicated EC is the fifth common cancer in both gender (22). Rafie manesh *et al.* by using join point analysis in central part of Iran for 2004-2008, indicated that common cancers for men were skin, stomach, colorectal, bladder, prostate and for women were breast, skin, colorectal, stomach and EC. The ASR of EC for women was estimated as 5 (23). 

**Table 2:** Comparison of EC ranking between 10 common cancers in Iran for 2004-2010

	Ranking	
year	women	men
2004	5	6
2005	5	6
2006	5	6
2007	5	7
2008	5	7
2009	5	8
2010	5	8

Mohammadi *et al.* in the study using join point regression, evaluated incidence of some common cancer for Iranian provinces and pointed that during 2002-2008, ASR of EC was increasing while, at 2008-2010 was decreasing (24). Somi *et al.* in a 5 years study found that the ASR of EC for women and men was 7.1 and 9.4, respectively (22). Meanwhile the worldly amount of ASR of EC for women and men was 10 and 10.4, respectively (25, 26). Esmaeilzadeh *et al.* calculated the ASR for EC and also mapped the results; based on this map, both Northeast and Northwest had high ASR (26). Present study showed ASR increased up to 2009 and decreased in 2010 for both genders. In developed countries, ASR is reported as 1.3, 6.8, in developing countries 6.5, 13.7 and in south and central Asia are 5.9, 8.1 for women and men, respectively (3). Comparing ASRs revealed that ASRs in Iran are higher than the developed countries and the EC incidence is increasing by time. Improving in cancer data registry program, could be a reason. 

 The studies on EC risk factors have identified Polycyclic Aromatic Hydrocarbons (PAHs), smoking, opium, hot tea and alcohol assumption are the main risk factors (27). Islami *et al.* in a study compared risk factors in Golestan (in the North) and denoted that EC incidence in men is more than women due to more consumption of smoke (28). Sadeghieh Ahari *et al.* for checking distribution of EC incidence in Ardabil, found that some factors such as low temperatures, rain fall, which results to increasing water nitrate, high red meat consumption and low sunshine duration, were effective in cancer incidence (29). Various studies have carried out on investigating EC incidence, but in Iran, there is not a comprehensive study to investigate risk factors covering all various climate aspects and such a study is suggested. 

Many studies focused on spatial distribution of gastrointestinal Cancer but evaluation of EC distribution in particular, has been done rarely before. Spatial analysis for EC in Iran is conducted for limited regions or some parts. Chamanpara *et al.* in their study for introducing high risk regions in north of Iran, by using shared component model, denoted that the highest RR is 1.5 and pointed that Northeast has the highest and Southern parts has the lower RR. They accentuate on the fruit and vegetable consumption as a preventive factor (10). Hosseintabar Marzoni *et al.* in a study in the North, by employing variables such as sex and age in an adjusted model, it has been concluded that for high-risk locations, RRs remain from 2004 to 2008 (11). 

To the best of our knowledge, all mentioned spatial researches were carried out at restricted Iranian areas, while present study is at national scale and for all provinces. The main goal of the present research is identifying high risk regions for EC by using spatial statistical method. Such a method in framework of Bayesian analysis and by regarding neighbor regions effects, will lead to advance of RR estimation (30). According to the results of present research, the provinces which are located in the North West, North and North East are as high risk regions. The RR estimates and also its spread for women was more than men. Previous studies revealed that Northeast and North parts of Iran are located on EC belt. This belt extends up to the central of Asia and China. In most countries which are on the belt (including Iran), EC incidence is high (31). Additionally, the distribution of EC in Iran with respect to other cancers has more dispersion (32) and the present maps may be helpful for research in the latter point. Our results suggest the role of exposure to environmental factors, climate variety and cultural food habitation in the EC distribution. We found that during the time, the range of RRs for women is less than men and decreased by time. For men also the RR range decreased but the RR variation is low in comparison with women. 

It is important to note, the maps showed the risk of EC is decreasing, but attention to high risk regions is a considerable fact for policy makers. The maps of present study, addition to introducing high risk places, may help to achieve new ideas in protective factors by comparing geographical situations in low and high risk regions. It is necessary to point that in recent years, coverage and quality of data registration in Iran is improving while, the misclassification problem in cancer data registration almost exist (33, 34). High or low appearance of cancer incidence may be as a result of improving in data registering program (35) and this subject may affect our results.

In conclusion, the Northern part of Iran is a high-risk area of the EC and should be given more attention in aspect of prevention programs, such as training on leaving the harmful food habits.
